# Cerebrolysin in Patients Diagnosed with Subarachnoid Hemorrhage—The Results of an Observational Cohort Study

**DOI:** 10.3390/biomedicines12112492

**Published:** 2024-10-30

**Authors:** Klaudyna Kojder, Konrad Jarosz, Agata Andrzejewska, Joanna Solek-Pastuszka, Karolina Skonieczna-Żydecka, Mariusz Kaczmarczyk, Anna Jurczak

**Affiliations:** 1 Anesthesiology and Intensive Care Department, Pomeranian Medical University, Rybacka 1 str., 70-204 Szczecin, Poland; konrad.jarosz@pum.edu.pl (K.J.); agata.andrzejewska@pum.edu.pl (A.A.); joanna.solek.pastuszka@pum.edu.pl (J.S.-P.); 2 Department of Biochemical Science, Pomeranian Medical University in Szczecin, Broniewskiego 24, 71-460 Szczecin, Poland; karzyd@pum.edu.pl (K.S.-Ż.); mariusz.kaczmarczyk@pum.edu.pl (M.K.); 3 Department of Specialist Nursery, Pomeranian Medical University, Zolnierska Str. 48, 71-210 Szczecin, Poland; anna.jurczak@pum.edu.pl

**Keywords:** SAH, Cerebrolysin, neuromonitoring

## Abstract

**Background:** Subarachnoid hemorrhage (SAH) is associated with high mortality and a high level of disability. Progress in surgical and endovascular techniques has lowered the mortality rate in patients with SAH. However, many patients are left with neurological impairment. **Objectives:** In our study we wanted to examine the impact of Cerebrolysin on treatment results in patients with SAH diagnosis. **Methods:** The data of 47 patients, divided into Cerebrolysin (26) and non-Cerebrolysin (21) group were included. We examined the correlation between Cerebrolysin administration and additional Amantadine treatment or neuromonitoring, craniectomy, and endovascular treatment and its impact on the Glasgow Outcome Scale (GOS) score, length of stay (LOS), and mortality. **Results:** Our study shows that Cerebrolysin improves the mortality rate in combination with neuromonitoring in a group of patients with severe SAH. It does not affect the raw values of GOS or LOS in patients with SAH. **Conclusions:** Further studies with larger patient groups are needed to investigate the role of Cerebrolysin as an additional treatment in SAH.

## 1. Introduction

Subarachnoid hemorrhage (SAH) is a life-threating condition associated with high morbidity and neurological impairment. The incidence of SAH worldwide is 6–8 cases per 100,000 per year [1]. We distinguish aneurysmal SAH (aSAH) and SAH developed after trauma (tSAH). The incidence of SAH differs per region, probably because of smoking habits, the prevalence and severity of hypertension, and genetic differences. Women have a 1.3 times higher risk of developing SAH than men, but the prevalence of unruptured aneurysms is twice as high in women. It was considered that female gender is an independent risk factor for aneurysm growth, but a recent meta-analysis could not confirm this hypothesis [2]. 

The risk factors for developing intracranial aneurysm are hypertension, smoking, and female gender. The risk factors for aneurysmal rupture and SAH are hypertension, older age, larger aneurysm size, posterior circulation aneurysm, and irregular aneurysm shape [2].

To calculate the 5-year risk of rupture of intracranial aneurysms found incidentally via imaging, the PHASE score was developed [3]. The score was derived from North American, European, Finnish, and Japanese populations. It may be misleading when calculating for other groups. The PHASE score takes into consideration six different points, as follows: population (North American and European; Finnish and Japanese), hypertension (Y/N), and age (<70 years, ≥70 years). 

Although mortality in SAH has lowered in the past 30 years, it is still very high. One quarter of patients die before reaching the hospital or emergency department [4]. It is also a serious burden to society as patients who survive SAH are often affected with disabilities. Twenty five percent of SAH survivors become reliant on others for their daily care [4]. Thirty five percent of patients report decreased quality of life, i.e., memory loss, depression, anxiety, and post-traumatic stress disorder [5].

The first sign of aneurysmal rupture and subarachnoid bleeding is a severe headache. It is often described by patients as the worst headache in their life. Symptoms of ruptured aneurysms may vary from only headaches and mild neurological deficits to severe neurological symptoms, including coma [5]. The Glasgow Coma Scale (GCS) is a routine part of aSAH patient assessment [6]. It provides an objective measurement of a patient’s level of consciousness and can quickly show if the patient’s condition deteriorates. Another scale used in patients with SAH is the GOS (Glasgow Outcome Scale) [7]. Cerebral imaging is a crucial part of aSAH diagnosis. The non-contrast computed tomography (CT) scan is still the most commonly used diagnostic tool, especially during the acute phase. Its sensitivity approximates 100% when it is performed up to 3 days after the aneurysmal rupture and SAH [8]. If a CT is negative or unavailable but there is still a strong suspicion of aSAH, a lumbar puncture (LP) may be considered. Xanthochromia, a yellow or pink discoloration of the CSF or red blood cells in the CSF, can be indicative of SAH, but also can be a result of a traumatic tap, limiting the diagnostic specificity of an LP. There are no definitive guidelines distinguishing a traumatic tap from an LP positive for SAH or red blood cell ratios for this method [9]. Other radiologic diagnostic tools are CT angiography (CTA) and magnetic resonance angiography (MRA).

There are multiple severity grading systems that help to assess patient prognosis and mortality risk. One of the most commonly used grading systems is the Hunt–Hess scale [10]. 

Delayed Cerebral Ischemia (DCI) is one of the causes of neurological worsening after SAH. DCI and rebleeding are the strongest predictors of in-hospital mortality and morbidity, and are associated with poor functional outcomes [11]. Usually, DCI occurs 3–14 days after the aneurysmal rupture and SAH. It has been associated at first with the vasospastic narrowing of the cerebral arteries, or so-called vasospasm (VSP). After analyzing over 230 publications regarding SAH, Dorsch et al. found that 43% of the total population of SAH patients had VSP confirmed with radiologic findings. The number rose to 67% when angiography was performed at exactly the time the VSP occurred [12]. Diagnosing DCI no longer requires the presence or occurrence of VSP during radiologic examination. The pathophysiology of DCI is complex and multifactorial, and its diagnosis is mostly based on clinical features and exclusion. On radiological findings, regions of impaired cerebral perfusion do not always correlate with the region perfused by the vasospastic artery. CT perfusion is increasingly used to diagnose VSP and DCI. It can determine if the spasm is severe enough to reduce blood flow and cerebral perfusion. Transcranial Doppler Ultrasound (TCD) has been quite commonly used, but it can only show changes in cerebral artery flow velocities, the pulsatility index, and whether the autoregulation of the cerebral arteries is preserved [11]. TCD is the most widely used screening tool because of its availability. It is non-invasive and can be performed as a bedside procedure and repeated as needed. TCD is the most sensitive for a middle cerebral artery spasm [13]. Numerous factors have been associated with the development of DCI. The risk factors for the development of DCI are a high-grade hemorrhage and a high Fisher score, as well as prolonged loss of consciousness [14]. The accurate prediction of DCI is important because high-risk patients may require intensive monitoring, including neuromonitoring, whereas low-risk patients may be safely discharged home earlier.

The most important part of the treatment is the neurosurgery or endovascular approach concerning the hemorrhages qualified for intervention. The other part of life-saving treatment is intensive care after the bleeding. This includes the following procedures: pharmacological coma, artificial ventilation, hemodynamics monitoring, and adjusting the mean arterial pressure according to threshold points. Concerning the pharmacological treatment, only Nimodipine, a calcium blocker, is included in guidelines as a pharmacological agent. Nimodipine delays, or helps avoid, DCI. Therefore, it also reduces mortality after SAH. Good or better results of Nimodipine use are well established in the literature, as was confirmed in a meta-analysis in 1996 (improved mortality, vasospasm, and rebleeding) [15]. The positive influence of Nimodipine on the outcome in patients with SAH was also confirmed by a recent meta-analysis in 2022. Hao et al. proved that the administration of Nimodipine positively correlates with lower mortality, better general outcome, and a lower incidence of DCI [16].

Another drug that has been investigated in the context of SAH is Cerebrolysin. Cerebrolysin is a mixture of neuropeptides of porcine origin, the neuroprotective properties of which have been shown in patients with stroke diagnosis. The meta-analysis from 2017 shows that Cerebrolysin affects the NIHSS score and mRankin score, which was especially emphasized in severe patients [17]. The meta-analysis from 2023 suggests that Cerebrolysin affects the mortality of SAH patients, and might affect the GOS and LOS score; however, more data are required [18]. 

In order to provide more clinical data, we have decided to conduct a study examining the possible influence of Cerebrolysin on the outcome of patients with SAH. The secondary outcomes stated were GOS, LOS, and mortality. In order to apply the multimodal approach to the treatment of acute neuro trauma, we also sought to establish the cross-talk between Cerebrolysin and other variables. We wanted to assess whether there is a correlation between Cerebrolysin administration and other aspects of treatment (Amantadine administration, additional neuromonitoring, craniectomy, or endovascular treatment) in obtaining different results according to GOS and LOS. 

## 2. Materials and Methods

### 2.1. Study Design 

Our study was a real-time, pre–post interventional experiment. This study was conducted without randomization, with a retrospective approach. The Clinical Trial Registration Number is NCT06429943. The trial was approved by the Pomeranian Medical University Bioethical Committee. As a primary outcome, we allocated study feasibility. Secondary outcomes included GOS, LOS, and mortality in patients diagnosed with SAH, treated with or without Cerebrolysin administration. 

### 2.2. Participants

This study was conducted in a population of patients diagnosed and treated at University Hospital No. 1 in Szczecin, Poland. The inclusion criteria were age > 18 years old, diagnosis of SAH, and treatment under ICU conditions. Patients with age < 18 years, a medical history of allergy to Cerebrolysin, and acute renal failure were excluded from this study. We also added to the exclusion criteria pregnancy, multi-organ trauma, and death within 48 h after admission.

### 2.3. Clinical Management

The patients were admitted to the hospital and initially assessed in the emergency room. After the radiological evaluation, they were qualified or disqualified for immediate neurosurgery or the neuroradiological procedure. All participants were evaluated according to GCS and divided into severe and non-severe groups. The patients with GCS scores 3–8 were included in the severe patients group, and patients with GCS scores 9–15 were included in the non-severe patients group. At the level of the emergency room, the following procedures were performed or continued: monitoring, intubation, sedation, MAP > 85 mmHg maintenance, head elevation, radiological assessment, neuro-consultation, and Cerebrolysin administration. After eventual surgical interventions all of the patients were admitted to the ICU department. In some of the patients, the surgery or endovascular treatment was scheduled on the next day after clinical status stabilization, according to the neurosurgeon or neuroradiologist’s qualification. At this stage, interventional treatment was initiated, as described in detail in Section 2.4. Some of the patients received Cerebrolysin at the dosage 30–50 mL as soon as possible, the latest being within 6 h after the hemorrhage. During the patients’ stay in the ICU, Cerebrolysin was continued. Patients were also additionally monitored with ICP direct measurement or indirect methods (NIRS, EEG, SjO2, or ONSD). Also, within the ICU, the standard procedures were provided (nutrition, infection prophylaxis). After ICU treatment, patients underwent initial rehabilitation, and then were transferred for further treatment to the neurosurgery or rehabilitation department.

**Figure 1:**
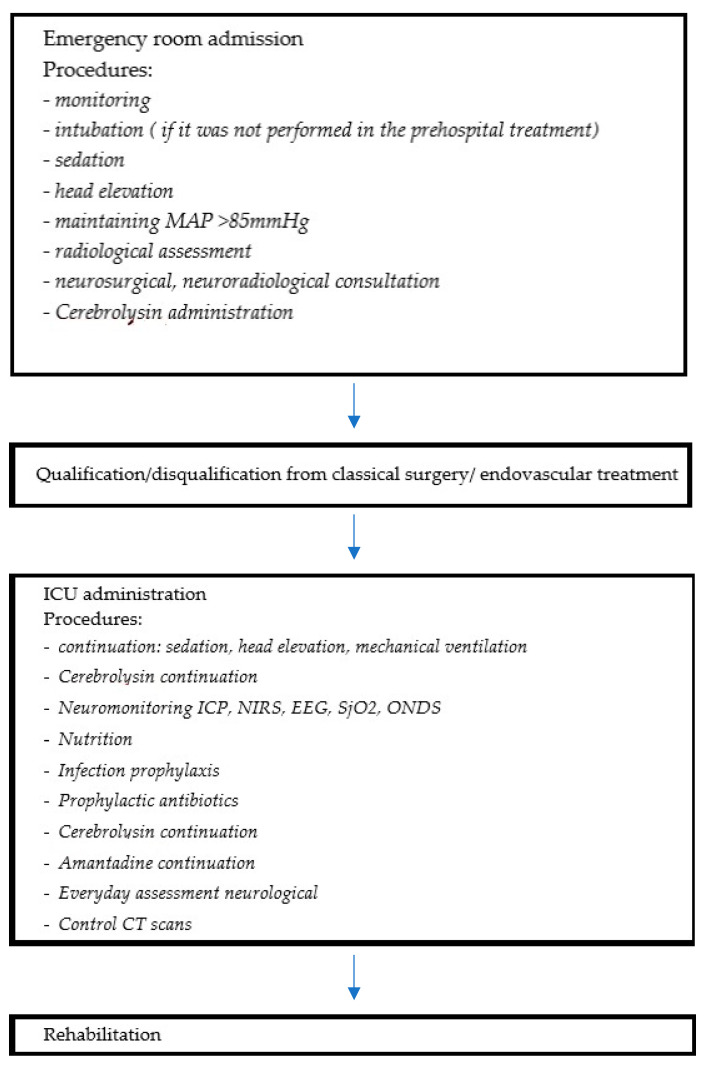

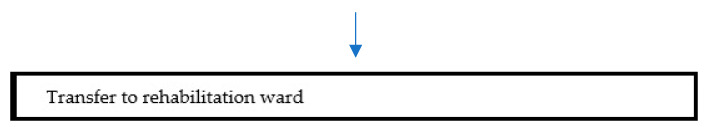
The route of the patient through hospital care, from emergency room to Intensive Care Unit and rehabilitation ward, with the procedures followed in each step.

The flowchart below illustrates the patients’ pathway in the hospital (Figure 1). 

Within the ICU, the patients were treated while following the general ICU and SAH guidelines according to Treggiari et al., “Guidelines for the Neurocritical Care Management of Aneurysmal Subarachnoid Hemorrhage” [19]. The patients were sedated with propofol and fentanyl in a continuous infusion with proper head elevation of at least 30°. Artificial ventilation with respect for lung-protective strategies was performed. To maintain the Cerebral Perfusion Pressure in patients monitored with an ICP direct catheter, the level of CPP > 60 mmHg was the target, and for other patients, a Mean Arterial Pressure (MAP) > 85 mmHg was required. Most of the patients required the continuous infusion of amino catecholamines to reach those targets. Direct ICP measurement or indirect methods using cerebral blood flow and pressure in the form of NIRS, EEG, SjO2, or ONDS were performed in some patients. From the day after initial admission, parenteral nutrition was introduced via nasogastric tube. 

### 2.4. Intervention

The internal standardized operating procedure for starting Cerebrolysin treatment was utilized. As per the intensive care physicians’ choice, Cerebrolysin at a daily dosage of 30 mL or 50 mL i.v. was initiated as soon as possible but no later than 6 h after the initial symptoms appeared. Cerebrolysin administration was continued during the ICU stay. 

### 2.5. Outcomes

As for the primary outcomes, study feasibility was stated, and the secondary outcomes were GOS, LOS, and mortality of the patients diagnosed with SAH [20]. 

### 2.6. Statistical Analysis

A statistically significant difference was established at two-sided *p* < 0.05. Continuous variables conforming to normal distribution have been expressed as mean +/− standard deviation (SD). Continuous variables that were not normally distributed have been presented as the median and interquartile range (IQR). Categorical data are expressed as numbers or percentages and were analyzed with the use of chi-square or Fisher’s test. The statistical analyses were performed with the use of Med Calc statistical software version 20.210. In the case of multiple variables, we used a generalized linear model with different families of distributions. The Gaussian family was used for the quantitative dependent variable (LOS), and the Poisson family for ordinal variables (GOS). For mortality calculations, the binomial family was used.

We conducted this analysis both with and without interaction terms involving SAH severity and other treatment factors, i.e., neuromonitoring or Amantadine administration. In those calculations, the “marginal effects” package version 0.15.1 in R was used. The effect sizes of Cohen’s d, Glass’s delta, and Hedges’ g were calculated. For technical operations, we used the 4.2.1 version programming language R (R Core Team, 2022). 

### 2.7. Ethical Statement

This research was carried out on the basis of decision number KB-0012/88/16, established by the Pomeranian Medical University Bioethical Committee. 

## 3. Results

### 3.1. Patient Characteristics

The data from 47 patients (23 females, 48.9%; 24 males, 51.1%) were analyzed. The mean age of the patients was 58.27 ± 13.76 (median, 58.0; IQR, 47.25–68.75). 

According to the HH scale, 16 patients were in good condition (33.3%), 29 were moderate (60%), and 3 were poor (6.2%). According to the GCS score, the patients were divided into 3 groups: mild trauma (GCS 13–15) was diagnosed in 8 patients (17%), moderate trauma (GCS 9–12) was diagnosed in 3 patients (6.4%), and severe trauma in 36 patients (76.6%). In order to divide the group into statistically justified groups, we divided the Cerebrolysin and no-Cerebrolysin groups into severe (n = 36; 76.6%) and non-severe (n = 11; 23.4%) subgroups. There were no differences in age or gender in the GCS subgroups (*p* = 0.6 and *p* = 0.81, respectively). The patient characteristics are described in Table 1 and Table 2 below.

The affected arteries were as follows: ACOA—n = 16 (34%), B—n = 3 (6.4%), ICA—n = 6 (12.8%), LMCA—n = 1 (2.1%), MCA—n = 6 (12.8%), non-aneurysmal SAH—n = 12 (25.5%), RVA, S 1—n = 1 (2.1%), and Sp—n = 1 (2.1%).

### 3.2. Patients Treatment

Cerebrolysin was administered to 26 (55.35%) of the patients enrolled, and Amantadine was administered to 17 patients (37.0%). Notably, data were not available for one patient. Embolization was performed in 23 individuals (48.9%), whilst additional neuromonitoring (NIRS, EEG, ICP, ONDS, and SJO2) was performed in 16 (34.8%). Classical aneurysm neurosurgery was performed in nine patients (18.8%). As shown in Table 3, no significant differences in SAH severity were detected in regard to these treatments. 

### 3.3. Cerebrolysin Efficacy

**Figure 2:**
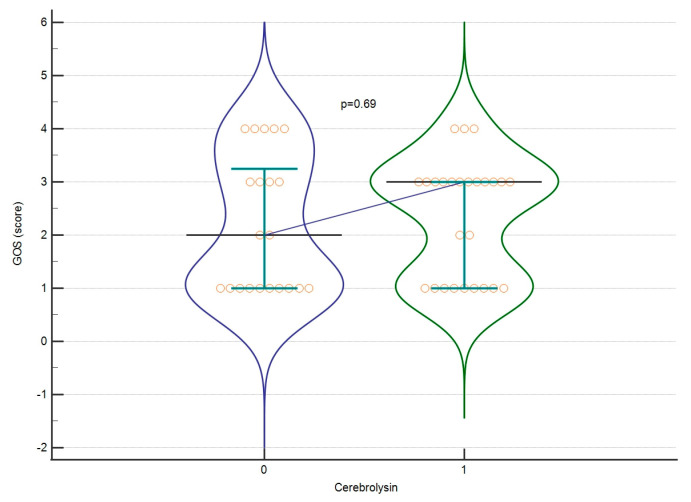
GOS regarding Cerebrolysin treatment. A violin plot depicting medians and IQR. Orange circles represent individual cases. The blue horizontal line connects medians.

**Figure 3:**
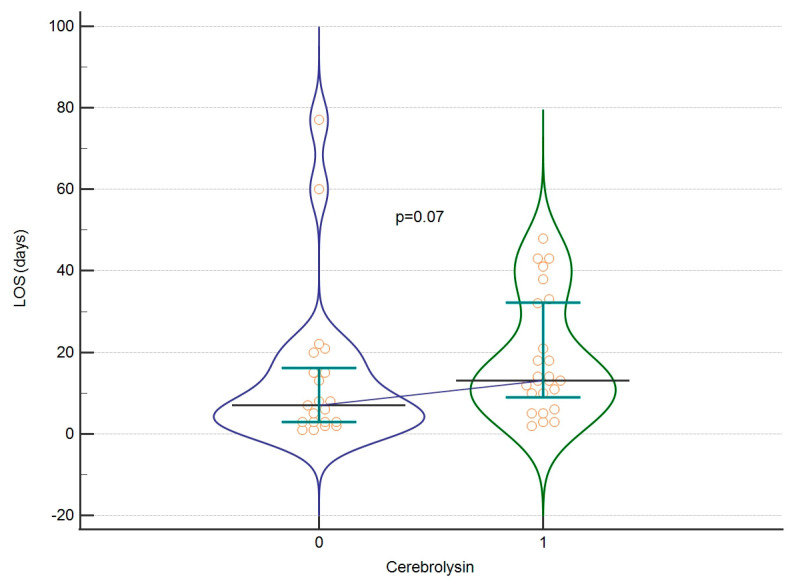
LOS regarding Cerebrolysin treatment. A violin plot depicting medians and IQRs. Orange circles represent individual cases. The blue horizontal line connects medians.

The mean GOS score for all patients classified as severe and non-severe was 2.27 ± 1.174. The mean score for LOS was 16.61 ± 16.91 days. At first, we assessed whether Cerebrolysin affects the raw value of GOS or LOS (severe and non-severe patients combined). We found no significant differences (*p* = 0.691 GOS, *p* = 0.07 LOS), as shown in Figure 2 and Figure 3. Afterwards, we assessed whether those parameters differ significantly when dividing the group into severe and non-severe subgroups according to GCS score. No significant differences were found in those calculations either. The effect sizes for these calculations are listed in Table 4.

We also employed generalized linear models (GLMs) to assess the effects of Cerebrolysin on the secondary outcomes (LOS, GOS, and mortality) with respect to other variables (i.e., age, gender, and treatment options) and interactions between them. Table 5 and Table 6 show details.

In our analysis, we did not find a statistically significant difference between patients treated with and without Cerebrolysin in terms of GOS or LOS scores. We also did not find a significant interaction with the use of Amantadine, performance of craniectomy or embolization, or additional neuromonitoring. Cerebrolysin also did not affect the mortality in severe or non-severe patients (data from 46 patients were available for this variable). Nevertheless, we found that severe patients subjected to neuromonitoring survive more frequently compared to patients without neuromonitoring. The data are presented in Table 7. 

When GLM was used, we found that Cerebrolysin’s effect on survival might vary depending on the application of the classical treatment. The use of Cerebrolysin was associated with a decrease in a probability of death when applying the classical treatment in parallel (estimate [Est.] = −0.4, SE = 0.22, *p* = 0.07). Details can be found in Table 8.

## 4. Discussion

In this study, we examined the effects of Cerebrolysin in patients with SAH on the following outcomes: GOS and LOS scores and mortality. We found that Cerebrolysin does not change the GOS or LOS of patients in this general study population. It also does not alter the GOS or LOS after division into the subgroups of severe and non-severe patients. Cerebrolysin did not change the mortality rate either. However, in our statistical analysis, we found that Cerebrolysin, when used with additional neuromonitoring in the group of severe patients (GCS < 8), is associated with a lower mortality rate. Cerebrolysin was provided at a dosage of 30–50 mL within the first 6 h after the onset of symptoms. The limitations of our study were the relatively small group of patients and the lack of blinding and randomization. 

In two other studies, Cerebrolysin was given at a dosage of 30 mL via intravenous administration [21,22]. In the work of Park et al., the authors concluded that Cerebrolysin affects mortality in poor-grade SAH patients (462 patients in total) [21]. Angiographic vasospasm occurred more often in the Cerebrolysin group; however, the modified Rankin Scale (mRS) scores were similar in both groups, and the mortality rate was significantly lower in the Cerebrolysin group. This could indicate that the main outcome did not only depend on the vasospasm [21]. In a study by Woo et al. (50 patients), the results for mortality and GOS were similar in the Cerebrolysin and non-Cerebrolysin groups [22]. The time of Cerebrolysin administration varied from 6 to 21 days in a meta-analysis from 2023 [18]. Our study’s conclusions are thus similar to the general trend seen in other reports on the efficacy of applying Cerebrolysin in SAH. We tried to focus not only on previously mentioned outcomes, i.e., GOS, LOS, and mortality, but also on the possible interaction between Cerebrolysin and other aspects of additional treatment. Therefore, we analyzed the use of Cerebrolysin in addition to Amantadine administration, and also neuromonitoring. We also examined the correlation between Cerebrolysin administration and neurosurgery or endovascular treatment according to GOS, LOS, and mortality. It seems important to note that applying Cerebrolysin may change the mortality rate of patients with severe SAH when used in combination with additional neuromonitoring. 

Patients with an SAH diagnosis constitute a more homogenous population than the population with stroke. Despite this prospective homogeneity, randomized trials including SAH treatment by use of neuroprotective drugs are still desired. From the onset of symptom appearance, every step on the way to recovery is crucial. Fast diagnosis in prehospital care, followed by fast transport to the facility, with options for specialized diagnosis and treatment options, are essential. Fast diagnosis and radiological assessment within the Emergency Room lead to appropriate decisions concerning surgical or endovascular gestures. Finally, for patients with a worse SAH grade, following standard procedures in the Intensive Care Unit with appropriate blood pressure (BP) thresholds and ventilation seems to be very important to survival and general outcomes. In the relevant literature, the multimodal approach is often mentioned [23]. It seems reasonable to assume that the general outcome is dependent on every step taken from the initial onset of symptoms. Neuromonitoring, early rehabilitation, and neuroprotection are also worth mentioning. 

In the pathology of SAH, morbidity is associated with the initial damage and its repercussions (hydrocephalus, hemorrhage, edema). The other factor associated with mortality is DCI. In the pathology of DCI, microvascular spasm and impaired interactions between glial and neuronal cells are mentioned [24]. In 2010, Laskowitz et al. noticed that symptoms associated with DCI were not associated directly with neuroimaging changes observed via angiography. The authors postulated that microvessel spasm could be responsible for the major effects of DCI [24]. On the other hand, the application of Nimodipine, associated with better functional outcomes, did not show any beneficial influence on radiological features. Magnesium influenced the occurrence of vessel spasm and the outcome. ET A (clozestan) and Nicardipine improved vasospasm; however, they did not affect the general outcome. The general outcome in patients with SAH seems to be influenced by a more complicated network of factors. DCI was originally linked to vasospasm [1940s], but further investigations revealed that the post-DCI infarct zones did not always correspond with previous detected vasospasms, according to radiological examination [11]. We could characterize DCI today as a temporally diffused process of brain tissue reacting to primary bleeding. It could manifest as vasospasm or infarct, but also via edema, blood–brain barrier (BBB) disruption, micro-thrombosis, the activation of the sympathetic nervous system, and inflammation [25]. Spreading depolarizations were also noticed in that period [25].

Taking the complex nature of SAH and the following DCI, it seems reasonable to examine the use of drugs with multipotential characteristics, such as Cerebrolysin, as an additional treatment. Twenty four years ago, Alvarez et al. described the neuroprotective properties of Cerebrolysin in vitro and in vivo [26]. A reduction in microglia super activity, which leads to overload with inflammation, was proven on glial cultures. The authors revealed the anti-inflammatory properties of Cerebrolysin, among other medications, on the extra production of proinflammatory cytokines (IL 1-beta) by microglia and the activation of glia cells (via the shape test) [26]. 

In 2020, Fiani et al. concluded in their analysis the multipotential role of Cerebrolysin. This drug is known for its interaction with GABAergic and cholinergic transmission [27]. On the cellular level, it acts on antiapoptotic paths, while on the other hand stimulating neurogenesis. With its mode of action that is similar to neurotrophic factors, Cerebrolysin creates antioxidative surroundings that enable neuronal restoration. Recent work also refers to it acting on the sonic hedgehog signaling pathways, which further helps to protect the neuronal cells from inflammatory responses [27].

**Figure 4:**
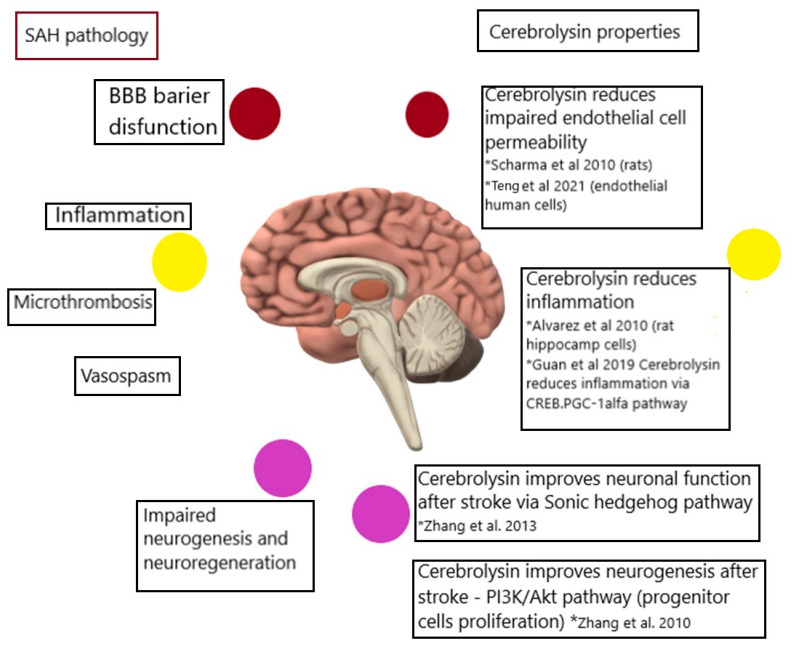
The possible ways through which Cerebrolysin acts on SAH pathology, including the reduction in impaired endothelial cell permeability (mentioned by Sharma et al., Hua et al.), which affects the BBB (blood–brain barrier). Cerebrolysin also reduces inflammation (mentioned by Alvarez et al., Guan et al.), and improves neuronal function and neurogenesis, affecting inflammation processes in SAH that impair neuro-regeneration (mentioned by Zhang et al.) The colored circles are provided to describe the pathogenesis and corresponding act of Cerebrolysin. * refers to the relevant publication [26,28,29,30,31,32].

In Figure 4, the possible ways through which Cerebrolysin acts on SAH are illustrated.

In the pathophysiology of SAH, after the initial primary damage, a period of possible DCI begins. In the pathophysiology of DCI, we can identify micro-vasospasms, vasospasms, impaired interstation between neuronal and glial cells, BBB disruption, micro-thrombosis, and inflammation. Cerebrolysin has been proven to influence impaired endothelial cell permeability [28,29,33,34]. It has also been observed to reduce neuroinflammation by reducing the proinflammatory cytokines and acting via the Creb/PGC1alfa pathway, inhibiting the inflammation process [29]. Cerebrolysin also improves the neuronal–glia cell crosstalk, acting through sonic hedgehog pathways [30]. Ultimately, it enhances neurogenesis following a stroke by acting on the PI3KAkt pathway [31]. To our knowledge, there are yet no descriptions of any activity of Cerebrolysin that could affect vessel spasm or act against micro thrombosis. However, all other points raised could indicate Cerebrolysin as an option for inclusion in additional therapy for patients with SAH. 

It seems that for complex diseases, such as SAH and others affecting the central nervous system, we must pursue a multitargeted approach. Aside from the standard treatment basis (diagnosis, transport, intervention, intensive care, and rehabilitation), there is a need to focus on the combination of additional aspects that can act together to offer the ultimate personalization of treatment for each patient. In our study, we attempted to assess the impacts of different aspects of hospital care for patients diagnosed with SAH. We analyzed the possible efficacy of Cerebrolysin in combination with other drugs, such as Amantadine, and also with classical neurosurgical and endovascular interventions. We did not find a statistically important correlation. This result also corresponds with those from previous studies [21,22]. 

In our study, the combined use of Cerebrolysin and neuromonitoring affected mortality in patients with severe SAH. This shows that enacting a multimodal treatment approach for a patient with SAH is reasonable and might lead to a better outcome. 

It thus seems that multimodal approaches to the treatment of patients with SAH represent an appropriate direction for future research. The complex pathogenesis of SAH may necessitate additive solutions acting at multiple target points. Perhaps a further direction would be to determine why Cerebrolysin is not as effective in SAH patients as in patients with a TBI diagnosis, and to define a personalized target group.

## 5. Conclusions

1. Regardless of SAH severity, Cerebrolysin does not affect GOS or LOS scores in our study.
2. Cerebrolysin does not affect GOS or LOS scores in combination with the use of Amantadine, craniectomy, embolization, or additional neuromonitoring in our study.
3. Cerebrolysin might affect mortality when combined with additional neuromonitoring in patients diagnosed with severe SAH in our study.

## Figures and Tables

**Table 1:** Patient characteristics: the group of patients included 23 females (49%), and the mean age was 58.27. Patients were assessed with the HH scale: 33% had a good HH grade, 60% were moderate, and 6% were poor.

Patients’ Characteristics	Female	Male	Age (Mean)	HH Good Grade	HH Moderate Grade	HH Poor Grade
n (%)	23 (49%)	24 (51%)	58.27	16 (33%)	29 (60%)	3 (6%)

**Table 2:** Patients’ characteristics: 8 patients were in the GCS 13–15 group, 3 patients were assessed as GCS 9–12, and 36 patients received <8 points. Further, 36 patients were placed in the severe group and 11 in the non-severe group.

GCS	GCS 13–15 Mild Trauma	GCS 9–12 Moderate	GCS <8 Mild	GCS Severe	GCS Non-Severe
n (%)	8 (17%)	3 (6.4%)	36 (76.6%)	36 (76.6%)	11 (23.4%)

**Table 3:** Treatment of SAH patients by SAH severity.

	Cerebrolysin		
GCS Qualitatively	NO	YES	p
not severe	5	6	0.95
severe	16	20	
	Amantadine		
GCS qualitatively	NO	YES	p
not severe	8	3	0.48
severe	22	14	
	Embolization		
GCS qualitatively	NO	YES	p
not severe	8	3	0.1
severe	16	20	
	Monitoring		
GCS qualitatively	NO	YES	p
not severe	6	5	0.4
severe	24	11	
	Classical Treatment		
GCS qualitatively	NO	YES	p
not severe	7	4	0.1
severe	31	5	

**Table 4:** Effect sizes of Cerebrolysin treatment by TBI severity.

	GOS			LOS		
	Cohen’s d	Glass’s Delta	Hedges’ g	Cohen’s d	Glass’s Delta	Hedges’ g
Severe	0.37	0.35	0.37	0.45	0.4	0.45
Not severe	0.69	1.09	0.67	0.19	0.16	0.2

**Table 5:** Cerebrolysin administration (Yes–No) based on GLM (Poisson family) for different sets of variables, including GOS as a dependent variable.

Predictor Space		Contrast Estimate	SE	*p*
Cerebrolysin ^1^ (YES vs. NO)		−0.15	0.54	0.78
Cerebrolysin (YES vs. NO) ^2^	Severe	0.4	0.47	0.39
	Not severe	−0.6	1.1	0.59
Cerebrolysin (YES vs. NO) ^3^	Amantadine (Yes)	−0.14	0.79	0.86
	Amantadine (No)	0.19	0.56	0.73
Cerebrolysin (YES vs. NO) ^4^	Classical treatment (Yes)	1.05	1.2	0.35
	Classical treatment (No)	−0.01	0.49	0.99
Cerebrolysin (YES vs. NO) ^5^	Neuromonitoring (Yes)	0.64	1.09	0.56
	Neuromonitoring (No)	−0.3	0.54	0.58
Cerebrolysin (YES vs. NO) ^6^	Embolization (Yes)	−0.09	0.65	0.89
Cerebrolysin (YES vs. NO) ^6^	Embolization (No)	0.38	0.6	0.53

^1^—age, gender, Cerebrolysin, Amantadine, Classical Tx, neuromonitoring, SAH severity (no interaction), ^2^—Cerebrolysin, SAH severity (interaction), ^3^—Cerebrolysin, Amantadine (interaction), ^4^—Cerebrolysin, Classical Tx (interaction), ^5^—Cerebrolysin, neuromonitoring (interaction), ^6^—Cerebrolysin, embolization (interaction).

**Table 6:** Cerebrolysin administration (Yes–No) based on GLM (Gaussian family) for different predictors, with LOS as a dependent variable.

Predictor Space		Contrast Estimate	SE	*p*
Cerebrolysin ^1^ (YES vs. NO)		−0.45	5.18	0.93
Cerebrolysin (YES vs. NO) ^2^	Severe	7.92	5.71	0.17
	Not severe	−3.9	10.3	0.71
Cerebrolysin (YES vs. NO) ^3^	Amantadine (Yes)	−5.68	7.03	0.42
	Amantadine (No)	7.22	5.15	0.16
Cerebrolysin (YES vs. NO) ^4^	Classical Tx (Yes)	−5.15	11.39	0.65
	Classical Tx (No)	7.85	5.58	0.16
Cerebrolysin (YES vs. NO) ^5^	Neuromonitoring (Yes)	−17.57	12.5	0.16
	Neuromonitoring (No)	7.15	6.26	0.25
Cerebrolysin (YES vs. NO) ^6^	Embolization	11.35	7	0.11
Cerebrolysin (YES vs. NO) ^6^	Embolization	−1.01	6.82	0.83

^1^—age, gender, Cerebrolysin, Amantadine, craniectomy, neuromonitoring, SAH severity (no interaction), ^2^—Cerebrolysin, SAH severity (interaction), ^3^—Cerebrolysin, Amantadine (interaction), ^4^—Cerebrolysin, Classical Tx (interaction), ^5^—Cerebrolysin, neuromonitoring (interaction), ^6^—Cerebrolysin, embolization (interaction).

**Table 7:** The mortality of SAH patients according to the severity of injury.

Variable	Whole Group			Severe			Not Severe		
Cerebrolysin	NO	YES	*p*	NO	YES	*p*	NO	YES	*p*
ALIVE	10	17	0.29	5	12	0.12	5	5	0.36
DEAD	10	9		10	8		0	1	
Amantadine									
ALIVE	15	12	0.1	8	9	0.06	7	3	0.54
DEAD	15	4		14	4		1	0	
Classical treatment									
ALIVE	20	7	0.2	14	3	0.59	6	4	0.45
DEAD	17	2		16	2		1	0	
Neuromonitoring									
ALIVE	14	12	0.08	8	8	0.04	6	4	0.27
DEAD	15	4		15	3		0	1	
Embolization									
ALIVE	13	14	0.77	6	11	0.39	7	3	0.54
DEAD	10	9		9	9		1	0	

**Table 8:** Cerebrolysin contrasts (Yes–No) based on generalized linear model (Binomial family) for different sets of predictors, with death as a dependent variable.

Predictor Space		Contrast Estimate	SE	*p*
Cerebrolysin ^1^ (YES vs. NO)		−0.03	0.14	0.81
Cerebrolysin (YES vs. NO) ^2^	Severe	−0.27	0.16	0.1
	Not severe	0.17	0.15	0.27
Cerebrolysin (YES vs. NO) ^3^	Amantadine (Yes)	0.07	0.22	0.74
	Amantadine (No)	−0.17	0.18	0.35
Cerebrolysin (YES vs. NO) ^4^	Classical Treatment (Yes)	−0.4	0.22	0.07
	Classical Treatment (No)	−0.12	0.17	0.45
Cerebrolysin (YES vs. NO) ^5^	Neuromonitoring (Yes)	−0.29	0.37	0.44
	Neuromonitoring (No)	0.04	0.19	0.81
Cerebrolysin (YES vs. NO) ^6^	Embolization (Yes)	−0.01	0.21	0.94
Cerebrolysin (YES vs. NO) ^6^	Embolization (No)	−0.29	0.2	0.15

^1^—age, gender, Cerebrolysin, Amantadine, craniectomy, neuromonitoring, and SAH severity (no interaction), ^2^—Cerebrolysin and SAH severity (interaction), ^3^—Cerebrolysin and Amantadine (interaction), ^4^—Cerebrolysin and craniectomy (interaction), ^5^—Cerebrolysin and neuromonitoring (interaction), and ^6^—Cerebrolysin and embolization (interaction).

## Data Availability

All the data available for sharing can be obtained by contacting the corresponding author.

## List of Abbreviations

SAH	subarachnoid hemorrhage
aSAH	aneurysmal SAH
tSAH	traumatic SAH
GCS	Glasgow Coma Scale
GOS	Glasgow Outcome Scale
CT	computed tomography
LP	lumbar puncture
CTA	CT angiography
CSF	cerebrospinal fluid
MRA	magnetic resonance angiography
VSP	vasospasm
DCI	Delayed Cerebral Ischemia
Length of Stay	LOS
TCD	Transcranial Doppler Ultrasound
ICU	Intensive Care Unit
NIRS	Near-infrared Spectroscopy
EEG	Electroencephalography
SjO2	jugular venous oxygen saturation
ONSD	Optic Nerve Sheath Diameter
